# Transcutaneous Auricular Vagus Nerve Stimulation Restores Cognitive Impairment in Morphine‐Withdrawn Rats: Role of BDNF and Glial Cells in the Hippocampus

**DOI:** 10.1111/adb.70016

**Published:** 2025-04-04

**Authors:** Somayeh Nazari, Saba Niknamfar, Hamed Ghazvini, Raheleh Rafaiee, Armin Allahverdy, Habibolah Khazaie, Seyedeh Masoumeh Seyedhosseini Tamijani

**Affiliations:** ^1^ Student Research Committee, School of Advanced Technologies in Medicine Mazandaran University of Medical Sciences Sari Iran; ^2^ Department of Neuroscience, School of Advanced Technologies in Medicine Mazandaran University of Medical Sciences Sari Iran; ^3^ Psychiatry and Behavioral Sciences Research Center, Addiction Institute Mazandaran University of Medical Sciences Sari Iran; ^4^ Neurodevelopmental Disorders Prevention Center Perinatal Institute, Cincinnati Children's Hospital Medical Center Cincinnati Ohio USA; ^5^ Department of Psychiatry, Faculty of Medicine Kermanshah University of Medical Sciences Kermanshah Iran

**Keywords:** BDNF, cognitive impairment, morphine, neuroinflammation, vagus nerve stimulation

## Abstract

Opioid use disorder (OUD) is a significant mental health problem, with prolonged usage potentially resulting in tolerance, addiction and cognitive decline, including learning and memory deficiency. At present, pharmacotherapy serves as the primary treatment approach for OUD. However, despite its status as a cornerstone of treatment, pharmacotherapy has certain limitations, thereby mandating the exploration of alternative modalities. This study evaluated the efficacy of transcutaneous auricular vagus nerve stimulation (taVNS) in multiple cognitive domains in morphine‐withdrawn rats. To induce morphine dependence, the rats were administered 10 mg/kg morphine for 10 consecutive days. taVNS was administered to the left ear of each rat and continued for 2 weeks. After electrical stimulation, various cognitive and emotional functions were assessed through related behavioural tasks, including open field, Y‐maze, novel object recognition and elevated plus maze tests. GFAP, Iba1 and BDNF expression levels in the hippocampus were determined via quantitative polymerase chain reaction (qPCR). Our investigation revealed that taVNS ameliorated the impairment of working and recognition memory induced by morphine in behavioural tests. Additionally, it exerts an anxiolytic effect. Moreover, taVNS counteracted the decreased concentration of brain‐derived neurotrophic factor (BDNF) and elevated levels of glial fibrillary acidic protein (GFAP) caused by morphine. Nonetheless, taVNS applied only at a frequency of 100 Hz has the potential to lower Iba1 levels independently of prior exposure to morphine. taVNS has been shown to exert a neuroprotective effect on morphine‐withdrawn rats. This outcome indicates that taVNS can be employed as a supplementary therapy with other pharmacological interventions for OUD.

## Graphical abstract

Transcutaneous auricular vagus nerve stimulation (taVNS) for 2 weeks at three different frequencies ameliorated the impairment of working and recognition memory induced by morphine. Additionally, it exerts an anxiolytic effect. Moreover, taVNS counteracted the decreased concentration of BDNF and elevated levels of GFAP caused by morphine. Nonetheless, taVNS applied only at a frequency of 100 Hz has the potential to lower Iba1 levels independently of prior exposure to morphine. taVNS has been shown to exert a neuroprotective effect on morphine‐withdrawn rats.

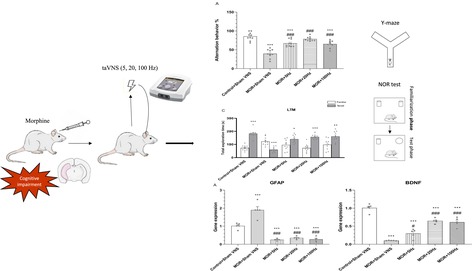

## 1 Introduction

Opioid use disorder (OUD) is a state marked by regular opioid consumption that is linked to a multitude of physical, psychological, societal and legal predicaments, resulting in substantial impairment or distress [1]. It was estimated that 60 million individuals were grappling with OUD on a global scale in 2022, leading to 71% disability and accounting for 69% of global overdose deaths [2]. Morphine is considered to be among the most commonly employed opioid analgesics in clinical practice. Although this drug has beneficial effects when it is administered in smaller doses and for shorter durations, repeated use may result in many adverse reactions. These may include addiction, withdrawal symptoms, memory impairment and immune system dysfunction. Studies have shown that individuals who misuse opioids experience elevated levels of microgliosis and astrogliosis markers, which are linked to changes in mood and memory [3, 4, 5, 6]. It leads to persistent changes in neuronal plasticity associated with the reorganization of patterns of synaptic connectivity in brain structures related to cognition and emotion, including the hippocampus and amygdala. These neuroadaptations are associated with the anxiety and drug‐seeking behaviour observed during morphine withdrawal. Hippocampal long‐term potentiation (LTP) is crucial for synaptic efficacy and plays an important role in learning and memory processes. Research has indicated that extended exposure to morphine has a negative effect on cognitive ability as a result of deficits in LTP, reduced hippocampal levels of brain‐derived neurotrophic growth factor (BDNF) and the induction of neuroinflammation [7, 8, 9, 10]. The hippocampus plays a key role in consolidating short‐term memory (STM) into long‐term memory (LTM) after training, and BDNF serves as a pivotal factor in the induction of LTP and, consequently, the formation of LTM [11].

Vagus nerve stimulation (VNS) is used for the management of depression and epilepsy in patients who do not respond to drugs [12, 13]. VNS can be either invasive or non‐invasive. Invasive VNS requires the placement of electrodes on the left cervical vagus nerve to stimulate it. However, its clinical implementation is somewhat restricted because of its invasive nature [14]. Transcutaneous auricular vagus nerve stimulation (taVNS) is a non‐invasive protocol and is a new form of VNS [15, 16, 17, 18, 19]. Currently, taVNS is under investigation for the treatment of cognitive disorders [20], anxiety [21], pain [22], alcohol dependency [23] and psychostimulant addiction [24, 25]. The auricular branch of the vagus nerve (ABVN) is a vagal branch that is distributed in the cymba concha, concha and tragus. The electrical stimulation of these regions results in changes in the activity of vagus nerve afferent fibres that convey some structures in the brainstem, such as the nucleus of the solitary tract, dorsal raphe nucleus and locus coeruleus (LC). Changes in the activity of the aforementioned areas can indirectly affect the functions of the hippocampus, amygdala and cortex, which are related to memory processes and emotion [21, 26, 27]. The frequency of stimulation emerges as a crucial element influencing the efficacy of the taVNS. Research has confirmed that different frequencies of electrical current stimulation can activate and produce different neurotransmitters within the brain. Preclinical studies have utilized a wide range of frequencies, showing neuroprotective effects on brain disorders. These studies suggest that the optimal frequency may vary depending on the type of disease [28, 29, 30]. Human and animal studies have substantiated the cognitive‐boosting effect of VNS [31, 32, 33, 34, 35, 36, 37]. Currently, research on the effectiveness of VNS on addiction is still in its early stages. The main aim of the present study was to evaluate and distinguish the effectiveness of three distinct taVNS frequencies, 5, 20 and 100 Hz, on various cognitive domains in morphine‐withdrawn rats via VNS and elucidate the mechanisms of action of taVNS in enhancing cognitive function.

## 2 Materials and Methods

### 2.1 Animals

Male Wistar rats weighing between 220 and 280 g, obtained from Sari, Iran, were housed under standard conditions. These conditions included an air‐conditioned room with a temperature of 22°C ± 2°C and a light–dark cycle of 12 h each. In addition, the rats had free access to clean water and food and were allowed to acclimatize to their new environment for 1°week. Also, the rats were handled for 5 min per day for 7 days to habituate them to human contact and manipulation by the experimenter before the start of the experimental schedule. The rats in groups of five were placed in plastic boxes with wood chip bedding. The experimental groups were kept and treated separately. All experimental procedures were granted approval by the Ethics Committee of Mazandaran University of Medical Sciences in accordance with the guidelines set forth by the Institutional Ethics, Animal Care and Use Committee (IR.MAZUMS.4.REC.1400.11710). The animals were randomly distributed into the following six groups:

1. Control + sham group: The rats received saline for 10 days and sham taVNS for 2 weeks.
2. Morphine group: The rats received morphine for 10 days and sham taVNS for 2 weeks.
3. Morphine + taVNS 5 Hz group: The rats received morphine for 10 days and taVNS 5 Hz for 2 weeks.
4. Morphine + taVNS 20 Hz group: The rats received morphine for 10 days and taVNS 20 Hz for 2 weeks.
5. Morphine + taVNS 100 Hz: The rats received morphine for 10 days and taVNS 100 Hz for 2 weeks.

### 2.2 Morphine Administration

**FIGURE 1:**
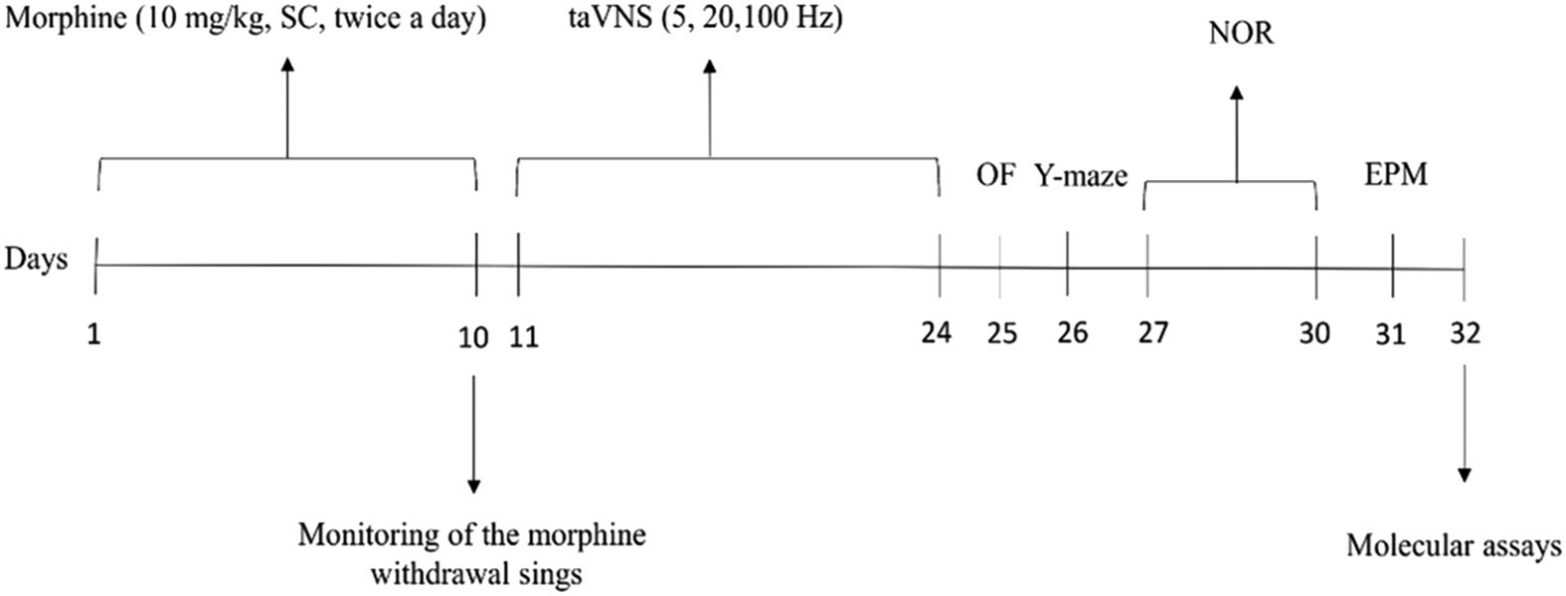
Experimental timeline.

Morphine sulfate (Temad Company in Tehran, Iran) was dissolved (10 mg/mL) in normal saline and then injected subcutaneously at a volume of 1 mL/kg twice daily, with injections given at 7:00 AM and 7:00 PM for 10 consecutive days. This particular morphine administration protocol is known to be sufficient for inducing dependence [38, 39, 40]. The control rats received the same treatment; however, instead of morphine injections, they were given injections of saline. Figure 1 presents a schematic diagram of the chronological sequence of events in the experiment.

### 2.3 Withdrawal Signs

After 10 days of morphine administration, we assessed withdrawal symptoms in two separate groups. We used the opioid antagonist naloxone (Daroo‐Pakhsh Co., Tehran, Iran) to confirm morphine dependence. The experimenter recorded the presence or absence of symptoms via observation. Intraperitoneal administration of naloxone at a dosage of 1 mg/kg was given to the rats 2 h after the final injection of morphine, and subsequently, the rats were placed in a transparent cage for 30 min and observed for morphine somatic withdrawal symptoms. The withdrawal symptoms included diarrhoea, ptosis, teeth chattering and writhing [41]. These groups did not undergo any behavioural or molecular tests, and they were only used to assess the development of morphine dependence.

### 2.4 taVNS

After 10 days of morphine injection, the three experimental groups of rats were subjected to taVNS for 14 consecutive days. The frequency of the taVNS stimulation varied for each group: 5, 20 and 100 Hz. The electrical stimulation was delivered by an electrical stimulator (model Novin 620P, Iran), and the pair of electrodes were fixed on the auricular concha region of the rats' left ear and secured with a clip to ensure the passage of electric current. Prior to fixing the electrodes, the stimulation region was cleaned with alcohol, and normal saline was used to facilitate electrical conduction All three experimental groups received the same taVNS parameters, including an intensity of 2 mA, stimulation for 30 s ON and 5 min OFF, with a total duration of 30 min and a pulse width of 500 μs [29, 33]. For the sham stimulation, electrodes were positioned on the margin of the left auricle, and stimulation was applied at a frequency of 20 Hz with the same taVNS stimulation parameters. The electrical stimulation parameters were monitored via an oscilloscope (model Hantek 6022BE, China). The taVNS procedures were administered daily between 9 and 10 AM.

### 2.5 Behavioural Tests

#### 2.5.1 Open Field (OF)

On the day following the cessation of taVNS (Day 25), the OF task was performed. An OF apparatus was constructed from Plexiglas measuring 60 × 60 × 30 cm, and its floor was divided into 16 uniform zones. The animal was positioned at the centre of the apparatus and allowed to freely search the open field (OF) arena for 5 min. The number of crossings, time spent in the centre area and distance travelled were documented via a video camera positioned above the OF apparatus and subsequently analysed by a blinded experimenter [42, 43].

#### 2.5.2 Y‐Maze

On Day 26, following the OF test, spatial working memory was measured via the examination of alternation behaviour in the Y‐maze test. This behaviour is motivated by the innate tendency of rodents to investigate previously unvisited environments. The Y‐maze task is dependent on the hippocampus, and alternation performance has been reported to be impaired in animal with lesions in the hippocampus [44]. The maze was constructed via Plexiglas (50 × 10 × 30 cm), and the arms were connected through a central area. At the beginning of the experiment, each rat was placed in the initial part of the starting arm (Arm A), whereas the guillotine door was closed. After 1 min, the guillotine door was removed, and the test time began. For 8 min, the arms that the animal entered were recorded in order (with the criterion that the base of the animal's tail had entered the arm). At the end of 8 min, the rat was removed from the device and returned to its cage. The video camera, positioned above the Y‐maze apparatus, was used to document the exploratory behaviour and then analysed offline by a blinded experimenter. Spontaneous alternation refers to successive entries into each arm of the Y‐maze, devoid of any repetitions. The percentage of spontaneous alternation was calculated as the ratio of actual to possible alternations, which is defined as (total number of arm entries − 2) × 100 [45].

#### 2.5.3 Novel Object Recognition (NOR) Test

The novel object recognition (NOR) test was developed to evaluate the STM and LTM of rodents and is based on their innate preference for novelty. This test was carried out over Days 27–30 and consisted of three phases: habituation, familiarization and the test phase. The test phase involves two steps: STM and LTM. This task was conducted in an OF with dimensions of 40 × 40 × 40 cm. Before the experiment was conducted, a control group of rats was used to assess the preference for each object, thereby ensuring the absence of any innate inclination towards any particular object. During the habituation phase, the rats were introduced to an OF arena for 10 min, without the presence of any objects, for 2 consecutive days. During the familiarization phase, two similar objects (A1 and A2) (heavy glasses bottle) were placed diagonally on the arena floor. The objects were randomized and distributed equally for all the animals. Each rat was given the opportunity to search for objects for 5 min. After each session, 70% ethanol was used to clean the chambers and objects. After the familiarization phase, a 90‐min interval elapsed, and the STM phase was initiated. This phase involved the assessment of two different objects (A and B), one being the familiar heavy glass bottle and the other being a toy. During this phase, the animals were given a time limit of 5 min to search for the objects. Finally, the LTM phase was performed after 24‐h intervals. The animals were given 5 min to explore two objects: a heavy glass bottle, which served as a familiar object, and a ceramic mug, which served as a novel object. A video camera positioned above the OF recorded the process of object exploration and was analysed offline by a blinded experimenter. The following criteria were used to define exploration: the length of time during which the rat directs its nose inside a 2 cm distance to the object or when it sniffs or paws the object [46, 47]. The difference in interactions between the new and old objects is used to assess memory, with the total exploratory activity evaluated by the total time spent visiting objects. The cut‐off time for adequate exploration time was established at 10 s for both objects per session. The hippocampus plays a crucial role in the process of object recognition memory, as it is responsible for encoding information related to the experience of objects, facilitating object memorization [48].

#### 2.5.4 Elevated Plus Maze (EPM)

Anxiety is commonly seen as a prominent symptom in the context of withdrawal syndrome related to a range of drugs that are frequently misused in both humans and rodents. On day 31, anxiety‐like behaviour was measured via the elevated plus maze (EPM) test, which is a widely validated test for measuring anxiety. The EPM apparatus comprised four arms with identical dimensions. Two of the arms were open, measuring 50 × 10 cm, whereas the other two were closed, having walls with a height of 40 cm. These arms were interconnected via a central square (10 × 10 cm). Each rat was positioned at the centre of the EPM and faced an open arm. The behaviour of the rat was observed for 5 min, and the video camera recorded the time spent and the number of entries into both the open and enclosed arms of the maze. The anxiolytic effect was demonstrated by the increase in either the amount of time spent in the open arms or the frequency of entry into the open arms [49].

After the rats completed the last behavioural test, they were deeply anaesthetized and then sacrificed. The hippocampal tissues were immediately isolated on ice, preserved in liquid nitrogen and stored at −80°C until the start of quantitative polymerase chain reaction (qPCR) assays.

### 2.6 qPCR

RNA was isolated from the hippocampus via TRIzol (Yekta Tajhiz Azma, Tehran, Iran). Briefly, the concentration and purity of the RNA samples were assessed using spectroscopy via a NanoDrop (Thermo Fisher Scientific, USA). The isolated RNA was subsequently stored in a freezer at −80°C. Following the equalization of the RNA concentration, the synthesis of cDNA was carried out via a cDNA synthesis kit (Parstoos, Tehran, Iran) in accordance with the relevant temperature cycle in the thermocycler. This investigation evaluated the mRNA expression of target genes (BDNF, Iba1 and GFAP) in relation to the beta‐actin reference gene. SYBR Green Master Mix (Ampliqon, Denmark) was used for real‐time PCR with the use of an ABI System (USA). The mRNA levels of the target genes were quantified via threshold cycles (Ct). The 2^−ΔΔCt^ method was applied to assess the mRNA levels of the target genes. The sequences of the primers are presented in Table 1 [46].

**TABLE 1:** Sequences of the primers used for qPCR.

Gene	Forward primer (5′–3′)	Reverse primer (5′–3′)
BDNF	GGCCCAACGAAGAAAACCAT	TTCCTCCAGCAGAAAGAGCA
Iba1	ATGCTGGAGAAACTTGGGGT	CAGTTGGCTTCTGGTGTTCT
GFAP	CTACATCGAGAAGGTCCGCT	GATTGTCCCTCTCCACCTCC
GAPDH	CAAGTTCAACGGCACAGTCA	CCCCATTTGATGTTAGCGGG

### 2.7 Data Analysis

The study reports data as mean ± SEM. Data analyses were conducted via the statistical software SPSS v22. GraphPad Prism software (Prism for Windows, Version 8.0) was used for graphing. The data from withdrawal symptoms were analysed non‐parametrically using the Fisher exact test. Paired *t*‐test was conducted for the familiarization phase and total exploration time in the NOR test. For analysis of Y‐maze, EPM and gene expression levels, one‐way ANOVA with Tukey's post hoc test was used to identify pairwise differences between groups, and to check data normality, a Shapiro–Wilk test was conducted. The significance level was regarded as *p* < 0.05.

## 3 Results

### 3.1 Assessment of Morphine Dependence

Withdrawal signs were measured by calculating the ratio of the number of subjects displaying withdrawal signs to the total number of subjects examined. As shown in Table 2, the administration of morphine resulted in physical dependence in the rats in the morphine group, as assessed through a specific range of behavioural symptoms including teeth chattering, diarrhoea, ptosis and writhing following naloxone administration, whereas the control group showed no signs of withdrawal.

**TABLE 2:** Withdrawal signs.

Groups	Somatic withdrawal symptoms			
	Teeth chatter	ptosis	Diarrhoea	Writhing
Control	0/9	0/9	0/9	0/9
Morphine	5/9*	8/9***	7/9**	6/9**

* *p* < 0.05 vs. control.

** *p* < 0.01 vs. control.

*** *p* < 0.001 vs. control.

### 3.2 The Effects of Three Different Frequencies of 5, 20 and 100 Hz taVNS on Locomotor Activity

**FIGURE 2:**
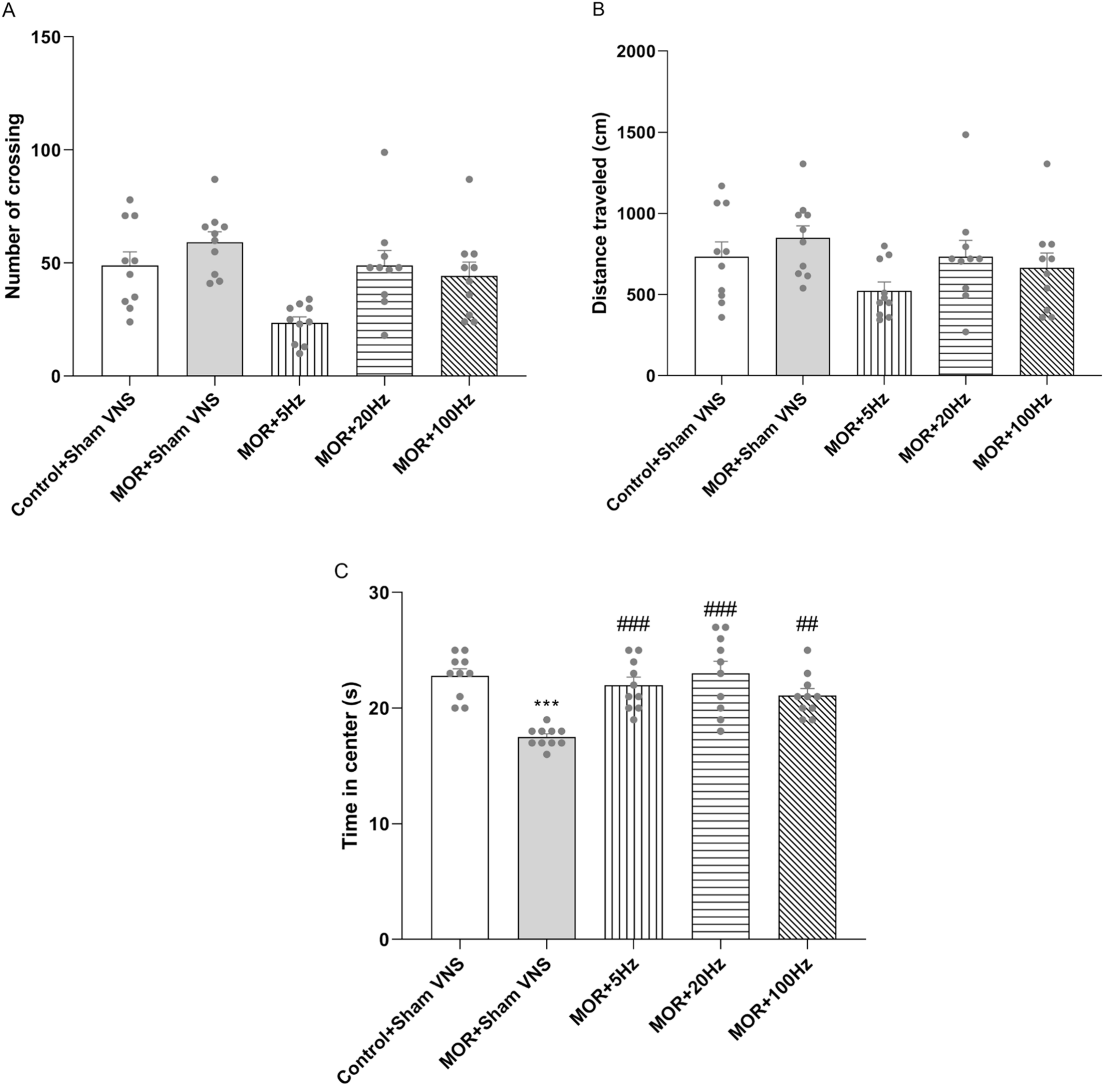
Effects of morphine (10 mg/kg) and different frequencies of taVNS (5, 20 or 100 Hz) on number of crossing (A), distance travelled (B) and time in centre of open field (C). The differences among all groups were determined via one‐way analysis of variance, followed by Tukey's test. The data are expressed as the mean ± SEM (*n* = 10). ***p < 0.001 vs. the control group; ###*p* < 0.001 and ##*p* < 0. 01 vs. morphine.

The locomotor activity index was calculated by counting the total number of crossings made by the subjects during 5 min in the OF arena, as shown in Figure 2A. After one‐way ANOVA, no significant differences were found among any of the groups in number of crossings (*F*_4,45_ = 1.085, *p* > 0.05) and distance travelled (*F*_4,45_ = 2.038, *p* > 0.05; Figure 2B). These findings suggest that the long‐term administration of morphine and taVNS at various frequencies did not affect locomotor activity. Moreover, Figure 2C showed a significant difference in time spent in the centre of the OF among the groups (*F*_4,45_ = 10.721, *p* < 0.001). Compared with the control group, the morphine group spent significantly less time spent in the centre (*p* < 0.001). Furthermore, there was a significant difference in the time spent in the centre between the morphine group and the taVNS groups at 5 Hz, 20 Hz (*p* < 0.001), and 100 Hz (*p* < 0.01). There were no significant differences between the taVNS groups and the control in time in the centre. Overall, these results suggest that taVNS reduced morphine‐induced anxiety behaviour.

### 3.3 The Effects of Three Different Frequencies of 5, 20 and 100 Hz taVNS on Spatial Working Memory

**FIGURE 3:**
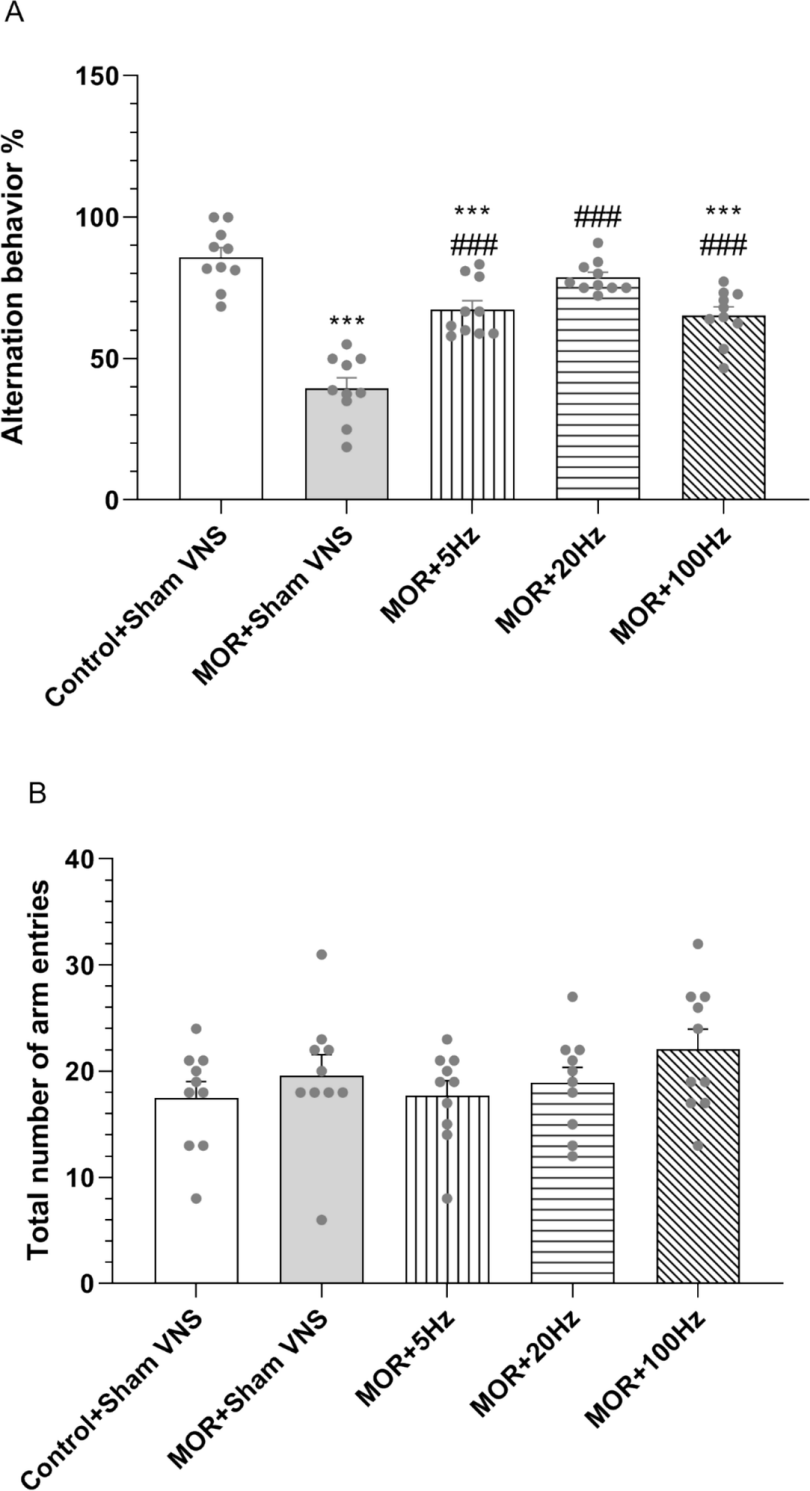
Effects of morphine (10 mg/kg) and different frequencies of taVNS (5, 20 or 100 Hz) on working memory. Alternation behaviour % (A) and total number of arm entries (B) were assessed in Y‐maze. The differences among all groups were determined via one‐way analysis of variance, followed by Tukey's test. The data are expressed as the mean ± SEM (*n* = 10). ***p < 0.001 vs. the control group; ###*p* < 0.001 vs. morphine.

The results of the one‐way ANOVA revealed significant differences in alternation behaviour between all the experimental groups (*F*_4,45_ = 33.535, *p* < 0.001). The data demonstrated that repeated administration of morphine had a notable detrimental effect on spontaneous alternation compared with that in the control group (*p* < 0.001), thus revealing a deficit in spatial working memory in the morphine group. The alternation score was significantly greater in the three different frequency groups treated with taVNS (5, 20 and 100 Hz) than in the morphine group (*p* < 0.001). There was also a significant difference in this score between the control group and the taVNS groups at 5 Hz and 100 Hz (*p* < 0.001). This suggests that taVNS partially improved the spatial working memory impairment caused by morphine (Figure 3A). In addition, the total number of arm entries was not significantly different among the experimental groups (Figure 3B).

### 3.4 The Effects of Three Different Frequencies of 5, 20 and 100 Hz taVNS on Recognition Memory

**FIGURE 4:**
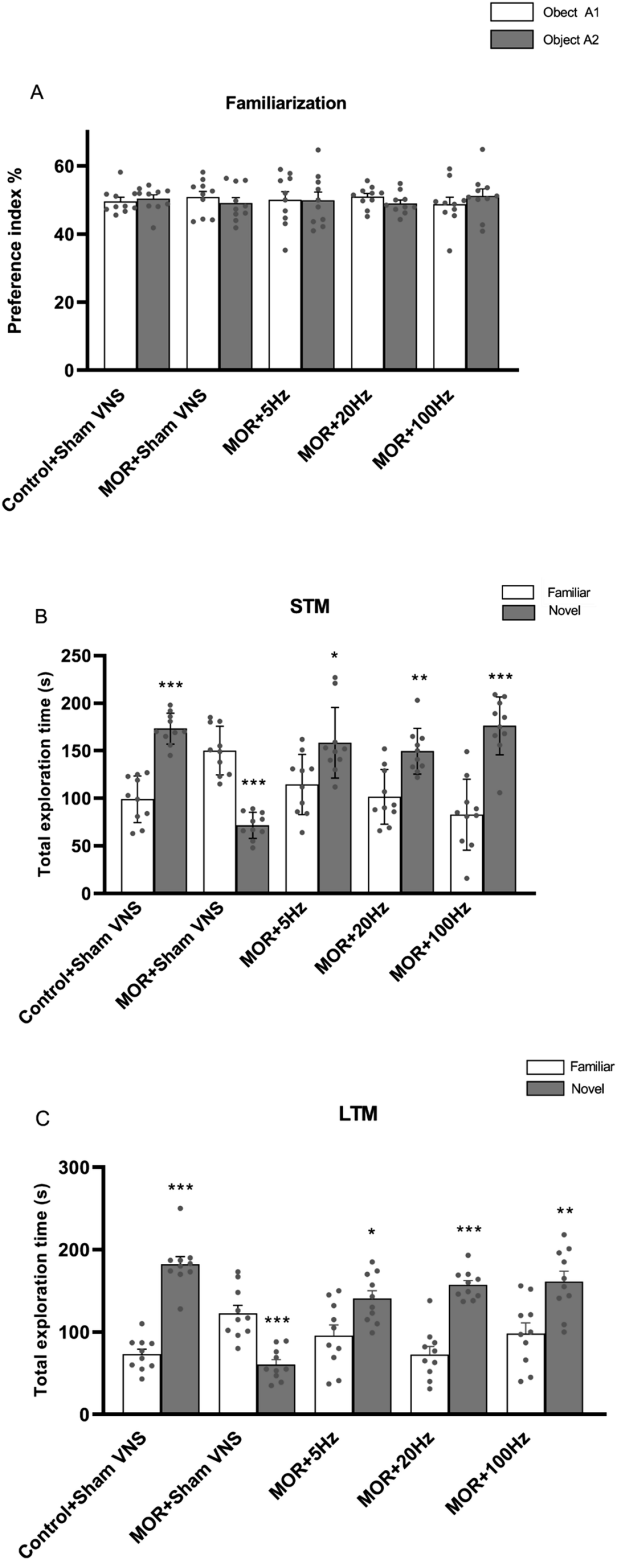
Effects of morphine (10 mg/kg) and different frequencies of taVNS (5, 20 or 100 Hz) on the preference index in the famillirization phase (A), total exploration time in the STM phase (B) and total exploration time in the LTM phase (C) were assessed in NOR test. The differences among all groups were determined via one‐way analysis of variance followed by Tukey's test. The data are expressed as the mean ± SEM (*n* = 10). *p < 0.05, **p < 0.01, ***p < 0.001.

In the familiarization step, the rats showed no biased exploratory preference in searching for both similar objects A1 and A2 equally, as shown in Figure 4A. During the STM step, *t*‐test analysis revealed that the control group spent more time exploring the novel object (*p* < 0.001) (Figure 4B). In contrast, the morphine group showed less interest in exploring a new object (*p* < 0.001), indicating STM impairment in the morphine group. Interestingly, the use of varying frequencies of taVNS significantly increased preference for the novel object, suggesting improved STM impairment caused by morphine (*p* < 0.05 for 5 Hz, *p* < 0.01 for 20 Hz, *p* < 0.001 for 100 Hz). In the LTM phase, the *t*‐test showed that rats in the control group had an increased preference for searching for new objects than familiar ones (Figure 4C). In contrast, the morphine group spent significantly more time on the familiar object (*p* < 0.001), indicating impaired LTM. Different taVNS frequencies (5, 20 and 100 Hz) led the rats to recognize the novel object and search significantly more for a new object (*p* < 0.05 for 5 Hz, *p* < 0.001 for 20 Hz, *p* < 0.01 for 100 Hz).

### 3.5 The Effects of Three Different Frequencies of 5, 20 and 100 Hz taVNS on Anxiety‐Like Behaviour

**FIGURE 5:**
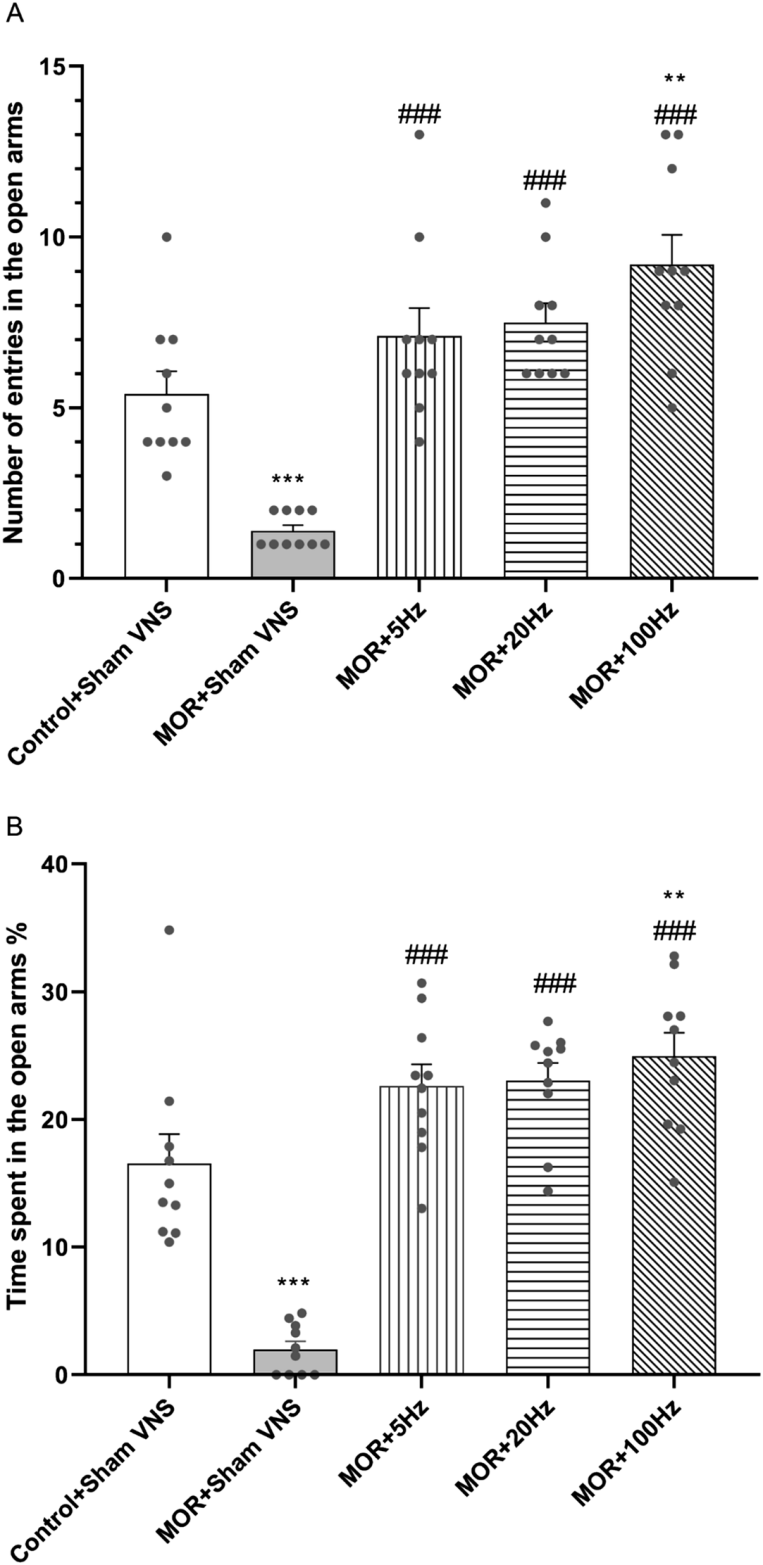
Effects of morphine (10 mg/kg) and different frequencies of taVNS (5, 20 or 100 Hz) on the number of entries in the open arms (A) and time spent in the open arms % (B) were assessed in EPM test. The differences among all groups were determined via one‐way analysis of variance, followed by Tukey's test. The data are expressed as the mean ± SEM (*n* = 10). **p < 0.001 and ***p < 0.001 vs control; ###*p* < 0.001 vs. morphine.

The results of one‐way ANOVA revealed significant differences in the number of entries into the open arms (*F*_4,45_ = 19.79, *p* < 0.001) (Figure 5A) and the percentage of time spent in the open arms (*F*_4,45_ = 31.85, *p* < 0.001) (Figure 5B) among all the experimental groups. The data, depicted in Figure 5, demonstrated that repeated administration of morphine resulted in notable anxiety‐like behaviour in comparison with that in the control group (*p* < 0.001). Compared with those in the morphine group, the anxiety‐like behaviour of the rats treated with different taVNS frequencies (5, 20 and 100 Hz) was significantly reduced (*p* < 0.001). Moreover, the number of entries into the open arms and percentage of time spent in the open arms were higher in the taVNS group at 100 Hz than control (*p* < 0.01).

### 3.6 Effects of Three Different Frequencies of 5, 20 and 100 Hz taVNS on the mRNA Levels of Astrogliosis (GFAP) and Microgliosis (Iba1) Factors in the Hippocampus

**FIGURE 6:**
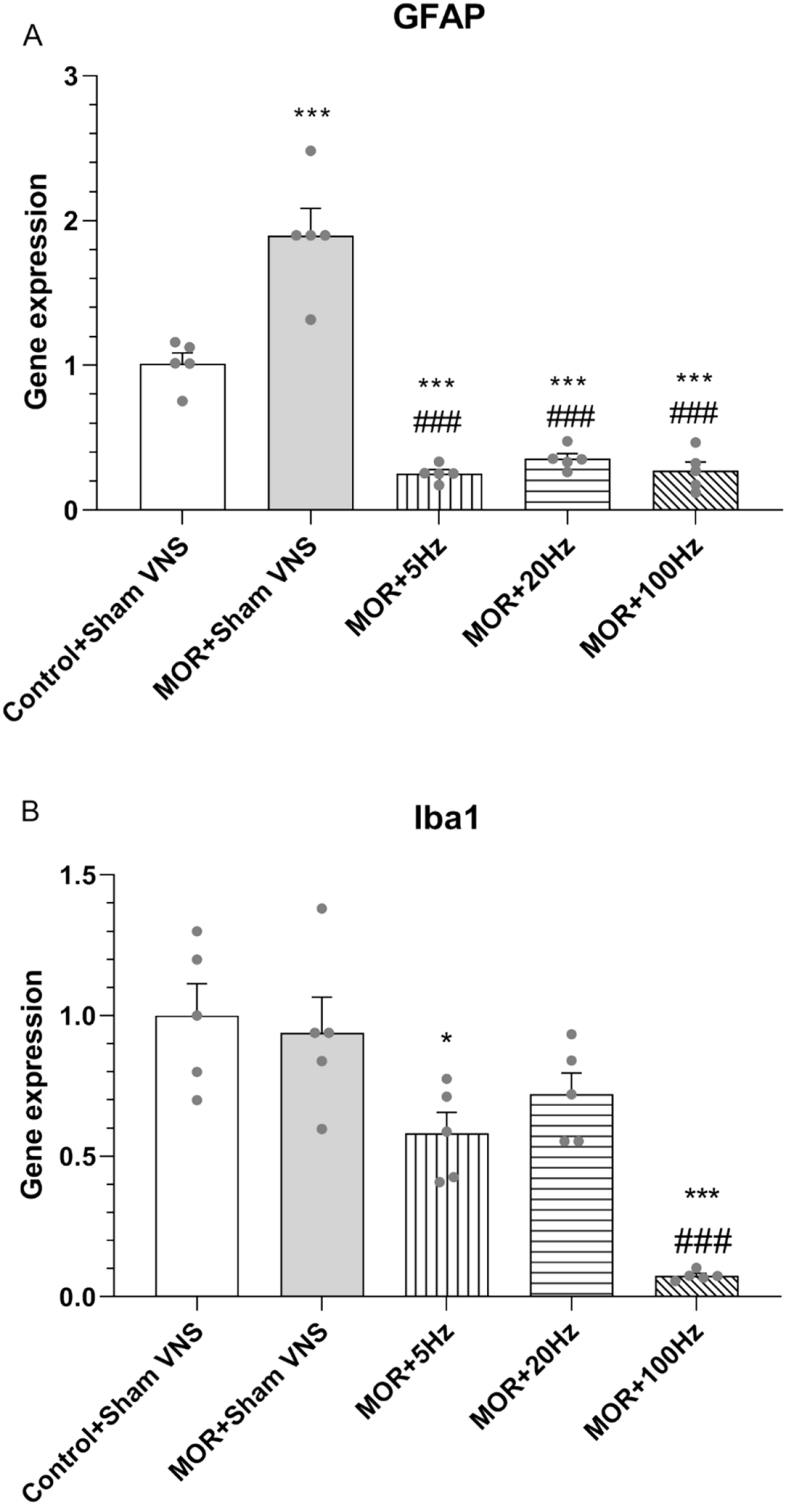
Effects of morphine (10 mg/kg) and different frequencies of taVNS (5, 20 or 100 Hz) on the gene expression of GFAP (A) and Iba1 (B). The differences among all groups were determined via one‐way analysis of variance, followed by Tukey's test. The data are expressed as the mean ± SEM (*n* = 5). *p < 0.05 and ***p < 0.001 vs. control; ###*p* < 0.001 vs. morphine.

The effects of treatment with different taVNS frequencies on the mRNA levels of GFAP and Iba1 in morphine‐treated rats were evaluated via qPCR. One‐way ANOVA revealed significant differences in GFAP (*F*_4,20_ = 56.586, *p* < 0.001) (Figure 6A) and Iba1 (*F*_4,20_ = 16.823, *p* < 0.001) (Figure 6B) between the experimental groups. The mRNA level of GFAP in the morphine group was significantly increased compared to the control group (*p* < 0.001). In addition, the mRNA level of GFAP was significantly lower in animals treated with 5, 20 and 100 Hz taVNS than in those treated with morphine (*p* < 0.001), whereas the mRNA level of Iba1 was significantly lower in animals treated with 100 Hz taVNS than in those in the morphine group (*p* < 0.001). However, there was no significant difference between the control and morphine groups. Additionally, expression of GFAB decreased in three different frequencies of taVNS groups compared with control (*p* < 0.001). The expression of Iba1 was statistically significant in the taVNS groups at 5 Hz (*p* < 0.05) and 100 Hz (*p* < 0.001) compared to control.

### 3.7 The Effects of Three Different Frequencies of 5, 20 and 100 Hz taVNS on the mRNA Level of BDNF in the Hippocampus

**FIGURE 7:**
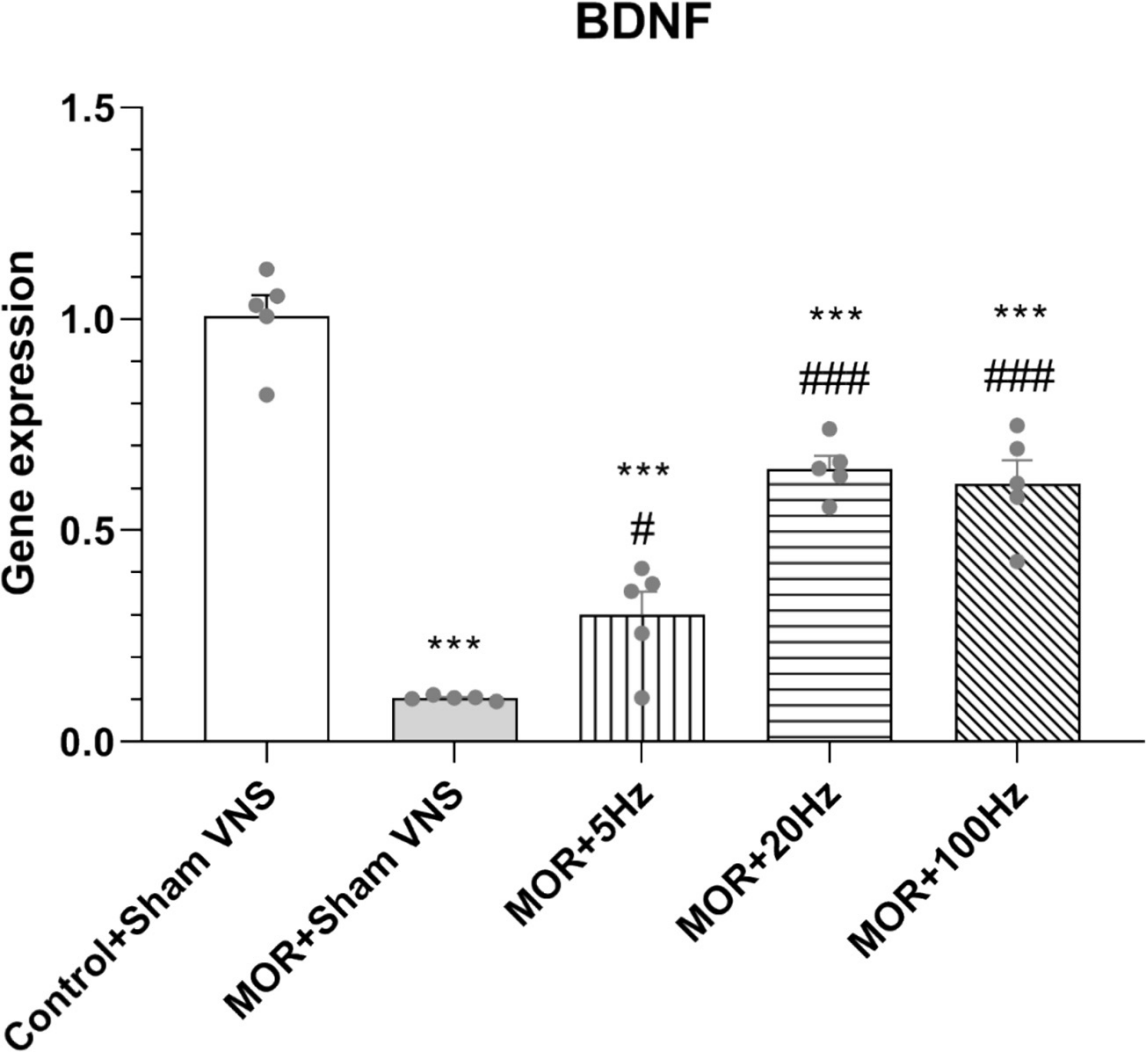
Effects of morphine (10 mg/kg) and different frequencies of taVNS (5, 20 or 100 Hz) on the gene expression of BDNF. The differences among all groups were determined via one‐way analysis of variance followed by Tukey's test. The data are expressed as the mean ± SEM (*n* = 5). ***p < 0.001 vs. control; #*p* < 0.05 vs. morphine; ###*p* < 0.001 vs. morphine.

The mRNA expression of BDNF was compared between groups that received different frequencies of taVNS via qPCR, and one‐way ANOVA revealed a significant difference (*F*_4,20_ = 63.612, *p* < 0.001). Compared with those in the control group, the gene expression of BDNF in the rats that were administered morphine was significantly lower (*p* < 0.001). However, treatment with taVNS at frequencies of 5 Hz (*p* < 0.05) and 20 Hz or 100 Hz (*p* < 0.001) significantly increased BDNF gene expression compared with that in the morphine group (Figure 7). A statistically significant difference was also observed between the control and taVNS groups in BDNF expression (*p* < 0.001), indicating that taVNS may partially counteract the decrease of BDNF in the hippocampus.

## 4 Discussion

Morphine is classified as a narcotic medication, and owing to its analgesic effects, it is widely prescribed for the management of postsurgical pain and cancer‐related pain. Chronic use of this drug can be associated with a high rate of abuse and can lead to dependence, tolerance and, eventually, addiction [50]. The aim of the current investigation was to evaluate the impact of taVNS intervention applied at three discrete frequencies on locomotor activity, memory, anxiety‐like behaviour and hippocampal gene expression of GFAP, Iba1 and BDNF in animals subjected to morphine. Our findings indicate that rats treated with morphine and subjected to taVNS at all three frequencies can be improved spatial working memory in the Y‐maze task as well as short‐term and long‐term recognition memory in the NOR task, both of which are known to be hippocampus dependent and also produced anxiolytic effects. Moreover, taVNS was related to decreased gene expression of GFAP, which are activity biomarkers of astrocytes, and compensated for the decrease in BDNF mRNA levels in morphine‐treated rats. Furthermore, the administration of naloxone to rats treated with morphine resulted in evident signs of withdrawal, which suggests that the repeated use of morphine in this manner can lead to dependency, a finding that is consistent with previous research [37, 40, 51]. In this study, there was no alteration in locomotor activity during the OF test. This observation suggests that the memory deficiency observed in behavioural tasks in rats subjected to morphine is not linked to locomotor function. Various studies have indicated that the effect of morphine on locomotor behaviour can be influenced by the dosage and time protocols employed [52, 53].

Many animal and human studies have reported that prolonged use of morphine leads to impaired learning and memory [54, 55, 56, 57]. Our research is in agreement with these findings and showed that animals subjected to morphine withdrawal presented a recognition memory deficit in the NOR test and working memory impairment in the Y‐maze task [58, 59]. The efficacy of VNS in enhancing synaptic plasticity and cognitive function has been demonstrated through animal research and clinical investigations in both healthy subjects and individuals with cognitive impairments [8, 35, 60, 61, 62, 63, 64, 65]. Clark et al. investigated the effect of VNS on different stages of memory for the first time. Research has shown that invasive stimulation of the vagus nerve can improve the consolidation and storage of memory in rats [66, 67], and other studies have shown that VNS enhances recognition memory in human subjects [68]. VNS is thought to modulate the production of several neurotransmitters that have the potential to reciprocally affect this phenomenon, such as the noradrenergic, serotoninergic, dopaminergic, cholinergic and GABAergic systems [20]. The induction of LTP in the dentate gyrus has been demonstrated via the application of VNS [60, 69]. This effect is hypothesized to involve the stimulation of noradrenergic receptors mediated by the LC. Anatomically, the vagus nerve does not possess a direct projection to the hippocampus. Thus, VNS indirectly affects hippocampal function via the LC. The vagus nerve sends sensory information to the solitary tract (NTS) in the medulla. In turn, afferent fibres from the NTS project directly to the LC. The LC then sends wide noradrenergic projections to different brain areas, including the hippocampus and cortex. The LC influences both neurons and glial cells within the hippocampus by releasing norepinephrine (NE), which directly binds to adrenergic receptors and initiates downstream cascades that activate synaptic efficacy [70]. Additionally, NE affects astrocytes and microglia through its interaction with the α2 and β1 adrenoreceptors, ultimately promoting the production of BDNF in these glial cells [71]. Various BDNF splice variants may be involved in learning and memory related to morphine addiction in the hippocampus [72]. The BDNF primer used in the present study targeted multiple splice variants, including X1, 10, 8, 9, 7, 6, 5, 4, 3, 2 and 1. Morphine interferes with the intracellular signalling pathways of BDNF, thus influencing its regulatory actions [73]. BDNF plays a crucial role in memory formation and maintenance by altering neuroplasticity and promoting neurogenesis in the hippocampus. The administration of morphine, on the other hand, can lead to detrimental changes in synaptic plasticity, including dendritic atrophy and reduction in the neurogenesis within the hippocampus and other limbic structures [74, 75]. Kaczmarczyk et al. reported that non‐invasive VNS may reverse chronic activation of microglia to a healthy state through the release of NE from the LC, which activates betareceptors on microglia in older APP/PS1 genotype mice [76]. Additionally, VNS induces memory consolidation through increasing the expression of BDNF and its receptor, tropomyosin receptor kinase B (TrkB), in the hippocampus [77]. In our investigation, morphine increased GFAP levels within the hippocampus but did not affect the expression of Iba1. Research on the effect of long‐term morphine administration on glial cell activity has shown inconsistencies. These variations may be due to differences in drug dosages, duration of administration, rat strains and the specific anatomical regions affected by morphine. For instance, in several studies, it has been shown that morphine triggers the activation of astrocytes and microglia in the ventral tegmental area [78, 79]. However, a survey conducted by Jokinen et al. observed that administering a 10 mg dose of morphine over 2 weeks did not lead to an increase in the expression of Iba1 in supraspinal regions, except for the substantia nigra [80]. In the present research, it is conceivable that the hippocampus may exhibit less susceptibility to this specific protocol of morphine administration in eliciting microglial activation. It has been identified that there are different active microglial phenotypes, such as M1 (pro‐inflammatory) and M2 (anti‐inflammatory). The study's limitation was that it utilized only a single marker to identify microgliosis. This approach may not fully capture the complexity of microglial cell activation, as they have different subpopulations and distinct functions. Therfore, we cannot be certain about the effect of morphine on microgliosis. In our study, we found that rats treated with taVNS at 100 Hz frequency showed a significant decrease in Iba1 levels compared to rats treated with morphine. There were no significant differences in Iba1 expression levels between rats administered morphine and control rats. The reduction in Iba1 expression after the administration of taVNS at 100 Hz frequency seems to be a direct consequence of its impact on Iba1 expression levels, regardless of exposure to morphine. Limited studies have investigated the link between cognition and the modulation of immune function through vagal mechanisms. Huffman et al. reported that percutaneous vagus nerve stimulation (pVNS), a minimally invasive technique, ameliorated memory dysfunction in lipopolysaccharide (LPS)‐treated mice by improving microglial morphology, reducing microglial activation (specifically Iba‐1 and CD68) and decreasing plasma levels of TNF‐α. Additionally, pVNS delivered at a frequency of 10 Hz resulted in better performance in a novel object recognition test than 20 Hz pVNS [8]. Further investigations are needed to fully elucidate the neurobiological mechanism underlying the anti‐inflammatory effects of VNS on the hippocampus of morphine‐treated rats. Previous studies have employed a broad spectrum of taVNS frequencies in their investigations. Wang et al. conducted a study to explore the potential antidepressant effects of three different frequencies: 5, 20 and 100 Hz. They studied the changes in behaviour and their impact on the hypothalamus–pituitary–adrenal (HPA) axis in rats with depression induced by chronic unpredictable mild stress (CUMS). The research findings indicated that, out of the three frequencies tested, 20 Hz seemed to be the most effective frequency for modulating the HPA axis function and producing an antidepressant effect. In contrast, neither 5 Hz nor 100 Hz has been shown to have any antidepressant effect [29]. This finding implies that the beneficial effects of taVNS may not necessarily be dose dependent. In alignment with these results, He et al. identified an antiepileptic effect associated with taVNS at 20 Hz in rats [81]. Furthermore, Yu et al. reported that a 40 Hz taVNS intervention suppressed the hippocampal P2X7R/NLRP3/caspase‐1 signalling pathway and ameliorated spatial learning and memory deficits in APP/PS1 mice [28]. Numerous studies have noted that taVNS interventions at frequencies of 2/15 Hz (alternating between 2 and 15 Hz every second) exhibit both antidiabetic and antidepressant properties [30, 82]. The literature describes various combinations of pulse width, frequency and stimulus intensity, and the effectiveness of these parameters may be influenced by different diseases. Studies suggest that optimal frequencies of taVNS can stimulate the central nervous system, particularly the central autonomic network, depending on specific disorders. Limited research has focused on determining the optimal frequency of taVNS for OUD. Therefore, we conducted a study to examine the neuroprotective effects of three taVNS frequencies on cognitive impairment in morphine‐withdrawn rats.

Several investigations have confirmed the positive effect of VNS in reducing anxiety symptoms [83, 84, 85, 86, 87]. We observed that VNS reduced anxiety‐like behaviour in morphine‐withdrawn rats. This was evidenced by decreased time spent in the centre zone of the OF and open arms of the EPM. It has been reported that VNS can alleviate anxiety and depression by increasing BDNF levels. Shah et al. reported that the use of K252a, an antagonist of tropomyosin receptor kinase B (TrkB), can eliminate the anxiolytic effects of VNS [88]. On the basis of the findings presented here, one may propose that the favourable effect of VNS on cognitive performance may be attributed in part to its impact on increasing BDNF levels and ameliorating anxiety. The impact of stress on the processes of learning and memory is intricate, with studies indicating both enhancing and hindering effects [89]. In rodents, the correlation between anxiety and memory is influenced by the duration of stress experienced. Evaluating the memory performance of animal models subjected to stress revealed that acute stress does not seem to affect LTM but does transiently disrupt STM. In contrast, chronic stress leads to the deterioration of both STM and LTM. The mechanisms through which stress influences learning and memory involve functional changes in brain regions crucial for cognitive functions, particularly the hippocampus [90]. Therefore, prolonged anxiety during withdrawal following chronic morphine exposure may result in memory impairment. Furthermore, in light of the extensive literature investigating the associations between the vagus nerve and anxiety [91], we hypothesized that taVNS would promote the activation of the parasympathetic nervous system, thereby reducing the overall perception of anxiety and enhancing cognitive performance.

Consequently, the relationship between anxiety, memory function and neuroinflammation in OUD is characterized by a bidirectional influence where neuroinflammation exacerbates cognitive deficits and anxiety and anxiety can contribute to continued opioid use and associated cognitive impairments. Understanding these interactions is crucial for developing integrated treatment approaches that address both the psychological and cognitive aspects of OUD.

Few investigations have been carried out to evaluate the effectiveness of VNS in the management of opioid addiction.

Yue et al. reported that pretreatment with iVNS and also taVNS have preventive effects on anxiety‐like behaviour, microglia activation and pro‐inflammatory cytokines upregulation in the hippocampus of mice subjected to heroin treatment [92]. Miranda and Taca reported that percutaneous aVNS (paVNS) administered over a period of 5 days is effective in significantly alleviating withdrawal symptoms in opioid abusers. This beneficial effect is attributed to the reduction in overactivation of the sympathetic system associated with withdrawal and the promotion of parasympathetic predominance modulated by the NTS [93]. Liu et al. reported that chronic VNS significantly prevents heroin‐seeking and relapse. It suggests VNS may help treat heroin addiction [94]. Jenkins et al. found that eight infants with neonatal opioid withdrawal syndrome (NOWS), who were more than 33 weeks old, received transcutaneous auricular neurostimulation (100 Hz, four times daily for 12 days). This treatment was shown to alleviate withdrawal symptoms in infants born with addiction [95]. Additionally, a clinical trial conducted by Gazi et al. confirmed that transcutaneous cervical vagal nerve stimulation (tcVNS) reduces opioid withdrawal symptoms in patients with OUD [96]. Driskill et al. shown that combination of 10 days of extinction training with VNS facilitated extinction and decreased drug‐seeking behaviour during reinstatement. In fact, rats that received VNS during an extinction period session had increased BDNF levels in the medial prefrontal cortex (mPFC) [97]. Taken together, these findings suggest that subsequent studies are warranted to clarify the neurobiological anti‐opioid mechanism associated with taVNS.

## 5 Conclusion

We concluded that taVNS improves various forms of memory function, such as working, short‐term and long‐term recognition memory, and has an anxiolytic effect on rats subjected to morphine. Furthermore, taVNS may partially counteract the decrease in BDNF and attenuate the increase in GFAP mRNA expression in the hippocampus. Nevertheless, further investigations utilizing other specific markers are imperative to clarify the precise mechanisms underlying the cognitive enhancement effects of taVNS. Given the favourable effects of taVNS as a non‐pharmacological intervention, which has minimal side effects, it can be implemented as a supplementary treatment in conjunction with other pharmacological therapies in the management of OUD.

## Author Contributions

S.M.S.T. supervised the study. S.N., S.N.F. and A.A. performed the animal experiments. R.R., H.G. and H.K. performed the data analysis and drafted the manuscript. S.M.S.T. revised the manuscript. All the authors read and approved the final manuscript.

## Ethics Statement

All experimental procedures were granted approval by the Ethics Committee of the Mazandaran University of Medical Sciences in accordance with the guidelines set forth by the Institutional Ethics, Animal Care and Use Committee (IR.MAZUMS.4.REC.1400.11710).

## Conflicts of Interest

The authors declare no conflicts of interest.

## Data Availability

The data that support the findings of this study are available from the corresponding author upon reasonable request.
